# Evaluations of retrospective frequency and phase correction methods for single-voxel MR spectroscopy at 7T

**DOI:** 10.64898/2026.09.21.753210

**Published:** 2026-09-22

**Authors:** Chu-Yu Lee, Jia Xu, Baolian Yang, Ralph Noeske, Vincent A Magnotta

## Abstract

Subject motion and gradient heating-induced frequency and phase offsets result in spectral misalignment during single-voxel MR spectroscopy (SVS) acquisitions. Several methods have been presented to align the spectra, but their performance on a 7T system is unclear. This study aimed to evaluate the practical importance of four retrospective correction methods, namely creatine fitting, residual water, spectral registration, and cross-correlation. SVS data were collected from 127 participants (88/39 female/male, 39±15 years) using a semi-localization by adiabatic selective refocusing sequence at 7T. Changes in the spectral linewidth, signal-to-noise ratio (SNR), similarity (mean similarity matrix (SI _mean_ )), and metabolite quantification (concentration, relative Cramer-Rao lower bounds (rCRLB)) after correction were evaluated. A Wilcoxon signed rank test was used to evaluate these changes. The *p* -values were adjusted using the false discovery rate ( *q* <0.05) for multiple comparisons. For significant changes, the effect size was further calculated using the Rosenthal formula. The results demonstrated that all correction methods resulted in a significant decrease in the spectral linewidth and significant increases in the spectral SNR and SI _mean_ . The mean changes by using the four methods were 0.76-0.99 Hz for the spectral linewidth change (effect sizes:0.73-0.86), 5.79-6.95 for the SNR change (effect size:0.60-0.72), and 0.03-0.03 for the SI _mean_ change (effect size:0.87-0.87). The correction also significantly increased the estimates of the metabolite concentrations of PCr, Gln, Glu, GPC, Ins, NAA, Scyllo, and Tau (effect size:0.25-0.57), and significantly decreased the rCRLBs (effect size:0.26-0.42) of Scyllo, Tau, and Gly. These results indicate that each method provides practical benefits in improving the spectral linewidth, SNR, and similarity by showing a strong to very strong effect of the improvements (effect size>0.60). The correction only showed a weak to moderate effect of the changes in the metabolite quantification (effect size<0.60), suggesting that whether the correction results in more reliable metabolite quantification requires further validation.

## Full Text Availability

The license terms selected by the author(s) for this preprint version do not permit archiving in PMC. The full text is available from the preprint server.

